# From Gene to Pathways: Understanding Novel Vps51 Variant and Its Cellular Consequences

**DOI:** 10.3390/ijms26125709

**Published:** 2025-06-14

**Authors:** Damla Aygun, Didem Yücel Yılmaz

**Affiliations:** Department of Pediatric Metabolism, Institute of Child Health, Faculty of Medicine, Hacettepe University, Ankara 06230, Turkey; aygun09@hacettepe.edu.tr

**Keywords:** proteomics, mitochondria–lysosome contact, GARP, EARP, vesicular traffic, autophagy

## Abstract

Disorders of vesicular trafficking and genetic defects in autophagy play a critical role in the development of metabolic and neurometabolic diseases. These processes govern intracellular transport and lysosomal degradation, thereby maintaining cellular homeostasis. In this article, we present two siblings with a novel homozygous variant in *VPS51* (Vacuolar protein sorting 51) gene (c.1511C>T; p.Thr504Met), exhibiting developmental delay, a thin corpus callosum, severe intellectual disability, epilepsy, microcephaly, hearing loss, and dysphagia. This study aimed to investigate the effects of the novel *VPS51* gene variation at the RNA and protein level in fibroblasts derived from patients. A comparative proteomic analysis, which has not been previously elucidated, was performed to identify uncharacterized proteins associated with vesicular trafficking. Furthermore, the impact of disrupted pathways on mitochondria–lysosome contact sites was assessed, offering a thorough pathophysiological evaluation of GARP/EARP (Golgi Associated Retrograde Protein / Endosome Associated Retrograde Protein) complex dysfunction. An analysis of mRNA expression indicated decreased levels of the *VPS51* gene, alongside modifications in the expression of autophagy-related genes (*LC3B*, *p62*, *RAB7A*, *TBC1D15*). Western blotting demonstrated a reduction in VPS51 and autophagy-related protein levels. Proteomic profiling revealed 585 differentially expressed proteins, indicating disruptions in vesicular trafficking, lysosomal function, and mitochondrial metabolism. Proteins involved in mitochondrial β-oxidation and oxidative phosphorylation exhibited downregulation, whereas pathways related to glycolysis and lipid synthesis showed upregulation. Live-cell confocal microscopy revealed a notable increase in mitochondria–lysosome contact sites in patient fibroblasts, suggesting that VPS51 protein dysfunction contributes to impaired organelle communication. The findings indicate that the novel *VPS51* gene variation influences intracellular transport, autophagy, and metabolic pathways, offering new insights into its involvement in neurometabolic disorders.

## 1. Introduction

Vesicular trafficking disorders, which ensure that proteins and other molecules produced within the cell are delivered to the appropriate target, and genetic defects detected in autophagy processes that cause lysosomal degradation of damaged proteins, organelles, cellular material, or macromolecules, are among the new and most remarkable metabolic/neurometabolic disease groups. Although the pathophysiology of diseases caused by the interactions of these two pathways, which are very important for cellular homeostasis, has been revealed in various diseases, the elucidation of their role in the pathophysiology of newly defined neurometabolic diseases are one of the most recent research topics [1,2].

The process of modification in the endoplasmic reticulum (ER), the delivery of molecules, such as proteins and lipids to other compartments and the plasma membrane, or the removal of various molecules from the plasma membrane and their subsequent transport to intracellular target compartments, is controlled by vesicular traffic. However, the return of membrane proteins from the Golgi apparatus to the ER and the return of various receptors to the Golgi or plasma membrane for use again (retrograde pathway) are also mediated by vesicular traffic [3,4]. The Golgi apparatus is key to the structure of eukaryotic cells and is made of flattened, perforated membrane stacks called cisternae. It is divided into three sections: cis, medial, and trans, where parallel cisternae are arranged in a stack. The cis-Golgi network (CGN), situated closest to the ER, is responsible for receiving ER-derived transport vesicles and returning ER-resident proteins to the ER [5,6]. The cisterna at the extreme position is continuous, with a tubular, branching, and reticulate compartment called the trans-Golgi network (TGN). The TGN is involved in the final stage of sorting, packaging, and delivery of most secretory proteins to their targets. Furthermore, cargo from early and late endosomes is retrogradely targeted to the TGN. When the endosome-mediated transport vesicle approaches the TGN, the transporter–Golgi interaction is mediated by tethering proteins, one of which is the GARP complex [7,8]. The GARP complex (a heterotetrameric binding complex) is composed of four subunits, namely VPS51, VPS52, VPS53, and VPS54. Defects in the GARP complex have been demonstrated to result in defects in both retrograde and anterograde trafficking. Studies in the “wobbler” mouse, considered a model for ALS, have shown that mutations in the gene encoding the *VPS54* subunit lead to a severe reduction in VPS54 levels and disrupt GARP complex assembly, thus causing spinal muscle atrophy. More recently, however, mutations in another GARP complex subunit, *VPS51*, have been associated with a neurodevelopmental disorder that causes lysosomal enlargement and impaired CI-M6PR (Cation-independent mannose-6-phosphate receptor) distribution, demonstrating the importance of this complex in cellular processes [9].

Lysosomal hydrolases are similarly glycosylated in the endoplasmic reticulum and transported to the Golgi, where they are prepared for transduction by the addition of mannose-6-phosphate (M6P) in the TGN. Here, they are recognized by M6P receptors (M6PR), packaged into the vesicle, and delivered to the lysosome [10,11,12]. Clathrin and COP coat proteins, GEF proteins, ARF, Sar1, SNAREs, VPS proteins, RAB proteins, microtubular structures, actin-binding proteins, molecular motor proteins, and cell membrane lipid regulators, which are necessary for the main stages of vesicular content (cargo) delivery, are encoded by more than a hundred genes and participate in the transport system [13,14]. In the transport system, in which all these proteins participate in the process, membrane–membrane interaction complexes are also involved in the delivery of lysosomal cargo through endosomes [15]. In this transduction pathway, membrane fusion complexes promote the attachment of endosome–lysosome and autophagosome–lysosome membranes mediated by Rab7. In this way, it also ensures the delivery of vesicle contents to the lysosome in the autophagic pathway [16,17,18]. Here, the GARP complex mediates the membrane fusion of recycling endosomes that retrograde transport M6P receptors to the trans-Golgi network for use again, which completes the transport of hydrolases to the lysosome [19,20].

In this article, we present two siblings with a novel homozygous variant in *VPS51* gene (c.1511C>T; p.Thr504Met), exhibiting developmental delay, thin corpus callosum, severe ID, epilepsy, microcephaly, hearing loss, and dysphagia. In the *VPS51* gene, a novel homozygous missense pathogenic variant c.1511C>T; p.Thr504Met is identified in two affected siblings by whole exome sequencing (WES) and confirmed by Sanger DNA sequencing.

Here, we aimed to determine the effects of the novel *VPS51* gene mutation at the RNA and protein level in fibroblast cells derived from patients. Also, the target protein(s) associated with vesicular traffic, which have not been previously elucidated by comparative proteome analysis, were investigated, as well as the link between autophagic flow and protein defects that provide intermembrane binding and fusion involved in vesicular traffic.

Our study leads to a better understanding of the molecular pathways leading to disease pathophysiology in vesicular traffic disorders, at least in part, serving as a prerequisite for the development of putative therapies. We believe that this study confirms the key role of the GARP and EARP complexes in normal autophagic flux, vesicular traffic, organelle–organelle interaction, and cellular homeostasis.

## 2. Results

**Figure 1:**
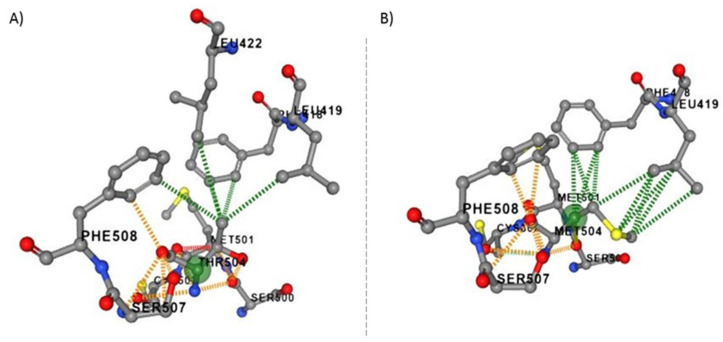
Surface structural detail of T504M for the wild type (**A**) and mutant type (**B**) VPS51 protein predicted by DynaMut2 software: (**A**) wild-type VPS51 protein; (**B**) mutant-type VPS51 protein (green signs have shown the alteration point).

DNA sequencing of the *VPS51* gene in all family members was shown, with results consistent with an autosomal recessive pattern of inheritance. DNA segregation analyses of novel missense variant NM_013265.4: c.1511C>T; p.Thr504Met in *VPS51* gene family members revealed that the affected siblings were homozygous, while parents and healthy siblings were heterozygous carriers for this nucleotide change.To predict the effect of p.Thr504Met, the novel variant, on VPS51 protein stability and dynamics, the wild-type and mutant-type predicted structural models generated by the DynaMut2 software (https://biosig.lab.uq.edu.au/dynamut2/, accessed on 1 June 2021) using AlphaFold database (https://alphafold.ebi.ac.uk/entry/Q9UID3, accessed on 1 June 2021) (structure: AF-Q9UID3-F1 PDB file) as the template structure are shown in (Figure 1A,B). Based on an in-silico analysis, the mutant protein is predicted to change the conformation and affect protein stabilization compared to the wild-type protein. The ΔΔG value of the c.1511C>T; p.Thr504Met variant (reported in this study) was calculated as −0.26 kcal/mol (destabilizing) (Table 1). According to the ACMG (American College of Medical Genetics) guidelines, the novel homozygous missense variant (NM_013265.4: c.1511C>T) in the *VPS51* gene was classified as a variant of uncertain significance (VUS). The potential pathogenic effect of this variant was predicted by an in-silico analysis program, Mutation Taster, which showed that the variant is assigned as a disease-causing pathogenic variation. The CADD analysis (CADD score: 25.6) and SIFT4G analysis (SIFT4G score: 0.01) for this variant suggest that it has damaging effects (Table 1).

**Figure 2:**
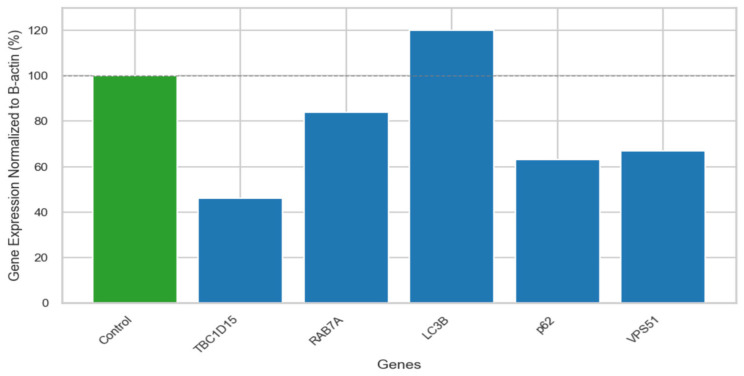
The mRNA levels of VPS51, p62, LC3B, RAB7A, TBC1D15 were assessed in patients and control by RT-PCR. β-actin was used as housekeeping gene.

We investigated the effect of the homozygous missense novel variant c.1511C>T; p.Thr504Met in the *VPS51* gene at the RNA/protein level and the relationship with autophagy. The patient and control samples were studied in duplicate, and the boxplot graph including the Ct values and mean values (used in 2^−∆∆Ct^ calculation) of each technical repetition is shown in Figure S1. Although a statistical test could not be performed due to insufficient sample size, the fold change comparison of the B-actin-normalized values of the patient and the control provided significant preliminary information for further functional studies. The mRNA expression levels of a disease-associated gene (*VPS51*), autophagosome-lysosome fusion related genes (*RAB7A*, *TBC1D15*), and autophagy pathway-related genes (*LC3B*, *p62*) were detected in this patient. Quantitative RT-PCR analysis has shown that the mRNA expression level of VPS51 (67%) was decreased in the patients compared to the control group (100%). Increased mRNA expressions of LC3B (169%) and decreased mRNA expressions of p62 (63%), RAB7A (84%), and TBC1D15 (46%) were observed compared to control group (100%) (Figure 2).

**Figure 3:**
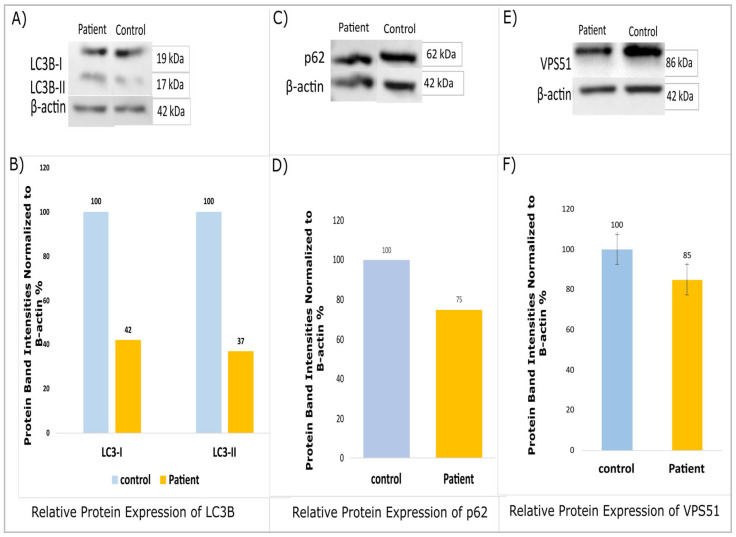
The *VPS51* mutation affects key proteins involved in autophagic flux. (**A**,**C**,**E**) The expression of total protein LC3B, p62, and VPS51 were assessed with the Western blot. β-actin was used as protein-loading control. (**B**,**D**,**F**) Bar graphs demonstrate a densitometry analysis of the Western blots normalized with β-actin using ImageJ 1.46v software.

A Western blot analysis revealed decreased protein expressions for LC3B, p62 (SQSTM1), and VPS51 in patients compared to the controls (Figure 3A–E). Furthermore, the statistical power of the study was found to be inadequate for the purpose of conducting the Western blot analysis. Protein samples were analyzed in duplicate (n = 2, technical replicates), and band intensities were determined using ImageJ 1.46 v software. The patient data were evaluated in terms of band intensity in comparison to the control group, and the fold change (patient/control ratio) of the values was normalized to β-actin. The findings presented are based on a single patient due to the rarity of this variant. It is evident that the results of this study require further validation through the execution of subsequent research projects, which should be conducted with larger cohorts.

### 2.1. Proteomic Profiling

The 1561 proteins identified in the three fractions were quantified using label-free quantification (LFQ) and converted into relative expression data in fibroblast cells from the patient and healthy individual (amplified from skin biopsy), resulting in expression profiles for all of the proteins. From the data, those identified by potential contamination, reverse effects, and proteins identified only at a single site were removed, and the analysis continued with 1498 proteins. LFQ intensities were filtered by Log2(x) transformation according to the valid value (minimum percentage of valid value is 70%), and those outside the normal distribution were removed. For the remaining 848 proteins, categorical annotation was performed by adding the UniProt Homo sapiens (UP000005640) annotation file. Statistically significant expression changes were determined via an ANOVA (analysis of variance) (*p* < 0.05), and then, for the remaining 585 protein expressions, a post hoc Tukey’s test (FDR < 0.05) was used to determine which sample was responsible for the expression change. For a total of 585 proteins, the fold change between the samples was determined, and a pathway analysis was performed with FC > 1.5 (upregulated) and FC < −1.5 (downregulated) (Tables S1 and S2). Of the 322 proteins, 132 were upregulated, and 190 were downregulated. These proteins were grouped according to whether they were upregulated or downregulated, and pathway analysis was performed [21,22].

**Figure 4:**
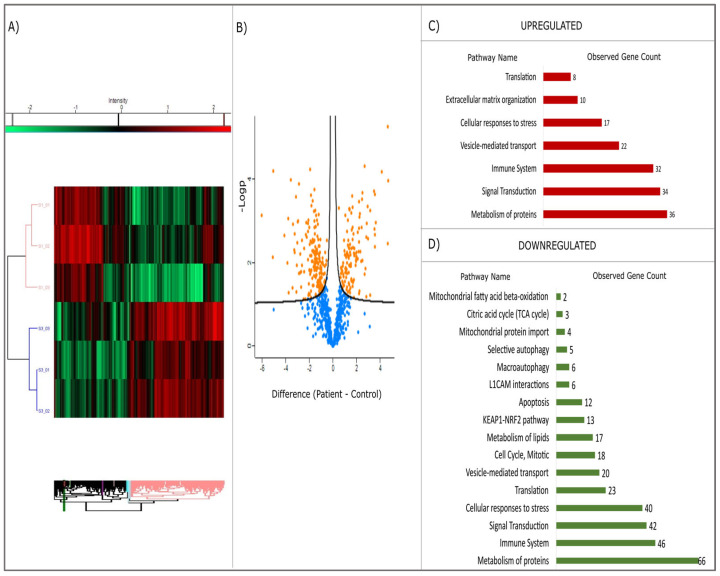
The results of the proteomic analysis are presented as a heatmap and a volcano plot. Green-to-red scale indicates low-to-high proteins expression levels (**A**,**B**). The molecular function of up-regulated and down-regulated pathways (**C**,**D**) in patients with *VPS51* mutation is demonstrated in the following images.

The heatmap, derived from the proteomic analysis (see Figure 4A), exhibits a clear distinction between the patient and control cohorts. The volcano plot (see Figure 4B) further elucidates the statistical significance of protein alterations (*p* < 0.05). In both graphs, the separation is evident, with the patient (S1) and healthy control (S3) group data distinctly separated. The dendrogram, which is a hierarchical cluster analysis, further demonstrates that the expression profiles of these two groups are different. In the patient group, some proteins demonstrated markedly increased expression (red), while others exhibited decreased expression (green). The volcano plot employs the Y-axis (−log10 *p*-value) to indicate statistical significance [23], providing a quantitative metric. Positively valued points (in orange) indicate proteins that are elevated in the patient group, whilst negatively valued points (in blue) indicate proteins that are decreased in the patient group.

Proteins involved in vesicular traffic (total: 22), protein metabolism (36), cellular response to stress (27), signal transduction (34), and immune system (32) pathways were found to be upregulated (Figure 4C), while proteins associated with protein metabolism (66), cellular stress response (40), KEAP1-NRF2 pathway (33), translation (23), L1CAM interactions (6), autophagy (11), apoptosis (12), and energy metabolism and mitotic activity (27) were found to be downregulated (Figure 4D). When the molecular functions of upregulated proteins are examined, it is seen that they are mostly grouped into protein binding, cell adhesion molecule binding, and cytoskeletal protein binding (Table S2). Downregulated proteins are mostly grouped into protein binding, catalytic activity, ion binding, hydrolase activity, enzyme binding, cell adhesion molecule binding, and GTPase activity (Table S3).

In order to comprehend the molecular adaptations that are induced by the *VPS51* gene mutation, a proteomic analysis was conducted with the objective of identifying differentially expressed proteins. The results of the analysis revealed that there were significant changes in several key metabolic pathways, including those relating to energy metabolism, lipid metabolism, and oxidative stress responses. Increased gene expression and pathway activations were observed, with proteins involved in glycolysis and lipid synthesis showing notable upregulation, suggesting a shift in the mechanisms of cellular energy production. Among the proteins that were found to be expressed at elevated levels were ACLY (ATP Citrate Lyase), which facilitates the conversion of citrate to acetyl-CoA, thus providing precursors for fatty acid synthesis; FASN (Fatty Acid Synthase), which was found to be critically elevated, indicating enhanced lipogenesis in response to the *VPS51* mutation; and PGK1 (Phosphoglycerate Kinase 1), a key glycolytic enzyme that is expressed at elevated levels to support ATP production under altered metabolic conditions. Finally, ATP5F1D (ATP Synthase Subunit D) has been identified as a key player in this process, with its upregulation suggesting a compensatory response to disrupted oxidative phosphorylation. Collectively, these findings underscore a metabolic reprogramming towards increased glycolysis and lipid synthesis, which may be linked to cellular energy demands and stress adaptation [24].

Conversely, a number of key enzymes implicated in mitochondrial energy metabolism and β-oxidation demonstrated a decline in expression, suggesting a decrease in reliance on oxidative phosphorylation and lipid degradation. ACADM (Acyl-CoA Dehydrogenase Medium Chain) and DECR1 (2,4-Dienoyl-CoA Reductase 1) are particularly noteworthy, as their reduced expression indicates suppressed mitochondrial β-oxidation. Downregulation of IDH1 affects NADPH production, potentially impairing antioxidant defenses and lipid metabolism. Reduced levels of CS (citrate synthase) and ACO2 (aconitase 2) suggest reduced flux through the TCA cycle. These changes imply a metabolic shift away from mitochondrial energy metabolism, which may contribute to the lipid accumulation observed in VPS51 mutant cells. Taken together, these findings suggest a dual metabolic adaptation—increased glycolysis and lipogenesis to support cellular energy requirements under stress. Reduced mitochondrial β-oxidation and TCA cycle activity likely contribute to energy conservation and reduction in reactive oxygen species (ROS).

Supplementary tables illustrate the pathways affected by these changes, while Figure 4C,D summarize the key up- and down-regulated proteins and their roles in cellular metabolism.

**Figure 5:**
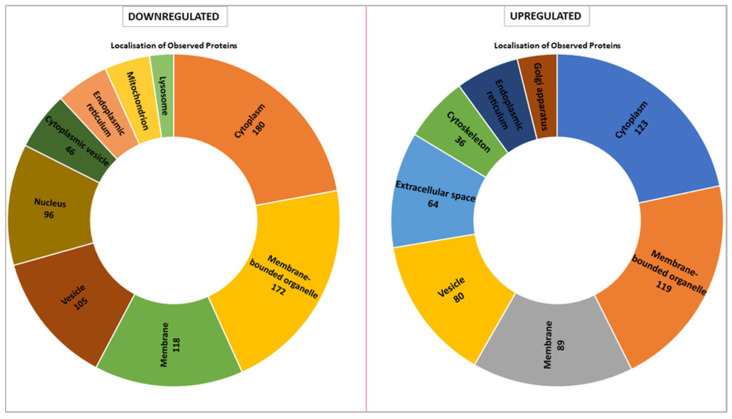
Cellular localization of upregulated and downregulated proteins in patient fibroblast cells.

The proteomic analysis reveals distinct localization patterns of upregulated and downregulated proteins in *VPS51*-mutant cells, with the majority of downregulated proteins being localized to the cytoplasm (180 proteins) and membrane-bounded organelles (172 proteins). Other major localizations include membrane-associated proteins (118 proteins), vesicle-related proteins (105 proteins), and nuclear proteins (96 proteins). In contrast, fewer proteins are localized to the cytoplasmic vesicle, endoplasmic reticulum, mitochondria, and lysosome compartments. Conversely, the majority of up-regulated proteins are predominantly localized to the cytoplasm (123 proteins) and membrane-bounded organelles (119 proteins). Notable increases are observed in membrane-associated proteins (89 proteins), vesicle-associated proteins (80 proteins), and proteins localized to the extracellular space (64 proteins). Proteins associated with the Golgi apparatus and cytoskeleton are also enriched but represent smaller proportions (Figure 5).

When we evaluated the intracellular location of proteins with expression differences between control and patient by proteomic analysis, we found that they were mostly membrane-bound organelles. Collectively and mostly, they were grouped in the cell membrane, vesicle, cytoplasm, and endoplasmic reticulum (Figure 5). In contrast, upregulated proteins were found in the Golgi, extracellular matrix, and cytoskeleton. The proteins localized in the Golgi were mostly proteins that stimulate the formation of Golgi stacks and ribbons and are involved in the transport and localization of molecules in both anterograde and retrograde pathways [25,26]. Upregulation of proteins involved in both structural and functional processes of the Golgi suggests a Golgi stress response. Similarly, some of the proteins localized in the extracellular matrix are involved in the cellular stress response (molecular functions have been shown in Table S4). Lysosome and mitochondria highlighted in downregulated proteins (Figure 5). In terms of molecular function, many proteins with lysosomal hydrolase activity are downregulated (Table S3), indicating that the lysosome cannot fulfill its normal function. However, the downregulation of proteins involved in mitochondrial pathways is not surprising considering the crosstalk between mitochondria and lysosomes. It is known that the function of these two organelles is coordinated, and loss of function in one leads to a secondary impairment in the other [27]. Our proteomic data support that the defect in lysosomal hydrolase delivery caused by the *VPS51* mutation disrupts the mitochondria–lysosome crosstalk, including the contact site. Taken together, increased RAB7A levels and decreased p62 and LC3 levels suggest proteasomal dysfunction and aggrephagy [28,29].

### 2.2. VPS51 Mutation Affects M-L Contact Sites

**Figure 6:**
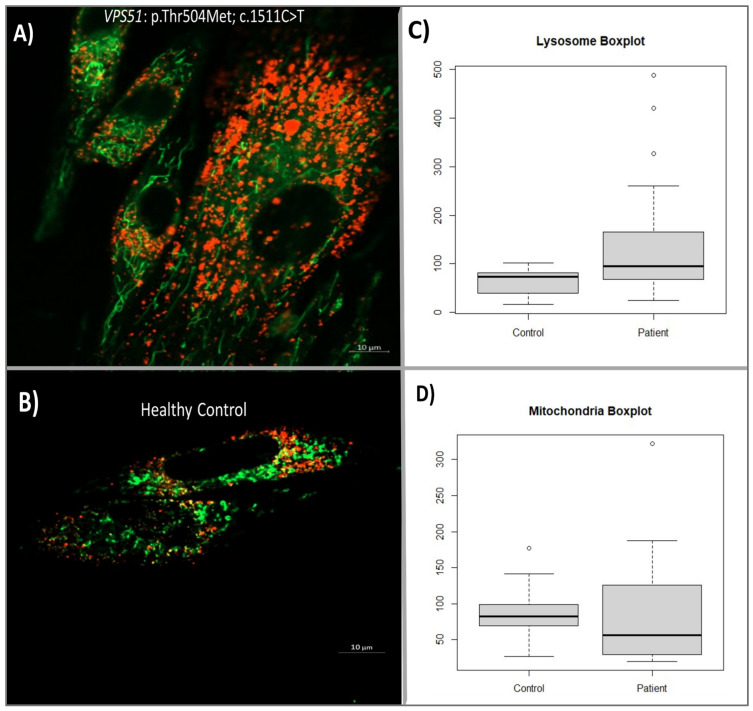
Confocal microscopy image of patient (**A**) and control (**B**) fibroblasts (Scale bar: 10 µm). Boxplot of normalized lysosome numbers (labeled with Lysotracker Red DND-99) (**C**) and mitochondria (labeled with Mitotracker Green FM) (**D**) numbers in cells.

We performed mitochondrial and lysosomal co-labeling and staining in patient and control fibroblasts using Lysotracker Red DND-99 (Invitrogen, Cat. No: L7528) and Mitotracker Green FM (Invitrogen, Cat. No:M7514). After the staining of the target organelles, live cell images were captured using a Zeiss LSM980 model confocal microscope (at Hacettepe University Advanced Technologies Application and Research Center) at ×63 objectives (Figure 6A,B). Twenty cell images from each group were included in the analysis and analyzed separately for mitochondria and lysosomes by using the Fiji-ImageJ program. Then, the distance and the number of surface connections between the labeled organelles in each cell were measured in terms of size and number. Statistical significance of patient and control data was tested using a Student’s *t*-test and Mann–Whitney U test according to the distribution patterns.

In this study comparing lysosomes in patient and control fibroblasts, it was found that there was a significant increase in the number of lysosomes in patient cells. The study measured the number of lysosomes and mitochondria separately in both groups and used a Shapiro–Wilk test to determine the normal distribution of the results. The difference between patients and controls was determined using the Student’s *T*-test, which revealed a 136.9% increase in lysosome numbers in patient cells compared to the control group (Figure 6C). Additionally, a Cohen’s d test confirmed that there was a large difference in lysosome data between patients and controls. Overall, this study suggests that lysosome numbers are significantly increased in patients with the *VPS51* mutation (n = 20, *p*-value: 0.0066).

We also compared the number of mitochondria in patient cells with the *VPS51* mutation to control cells and found that there was no significant increase in the number of mitochondria in patient cells. The significance of the difference was determined using the Student’s *t*-test, which showed that the slight difference in the number of mitochondria in the patient cells compared to the control was not significant (Figure 6D).

**Figure 7:**
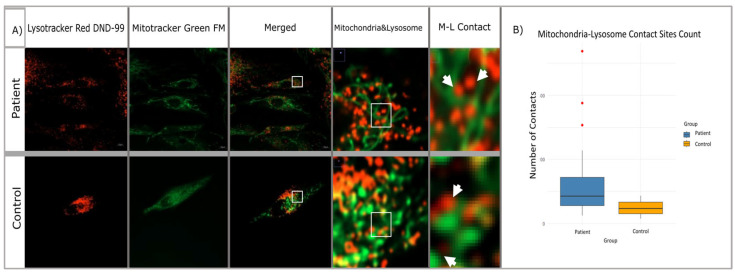
Confocal microscope image of mitochondria (**A**) (labeled mitotracker green), lysosomes (labeled lysotracker red), and contact zones in *VPS51*:pThr504Met; c.1511C>T mutated patient and healthy control cells (**A**). Boxplot of mitochondria–lysosome contact sites count for patient and control (**B**). Scale: 10 µm, 0.5 µm, and 0.1 µm; white arrows indicate mitochondria–lysosome contact zone (distance < 10 nm).

Confocal microscope images obtained from patient and control fibroblasts (n = 20) were examined to investigate the potential impact of mutations in the membrane tethering complex subunits on the mitochondria–lysosome contact sites in the cell (see Figure 7A). The DiAna plugin of the Fiji-ImageJ program was utilized to determine the organelles in contact between the mitochondria and lysosomes within the cells, with a maximum distance of 10 nm. It is possible that there may be differences in size, number of organelles, or structural differences between cells. Therefore, the contact numbers obtained were normalized to the number of mitochondria and lysosomes in each cell. This approach was adopted to mitigate the impact of these variations on the cellular level. Statistical significance tests were conducted, similar to the approach employed for mitochondria and lysosome numbers. The results of the Shapiro–Wilk test suggested that the data of both groups might be following a normal distribution. Therefore, it was decided to use the Student’s t-test to determine whether there was a difference between the number of contacts. The analysis revealed that the mean of the control group was notably lower than the mean of the patient group, and the normalized number of M-L contacts in the patient group increased by approximately 72.8% compared to the control group (Figure 7B). This variation was found to be statistically significant and not merely random (*p* = 1.4807 × 10^−8^).

## 3. Discussion

Disorders associated with vesicular trafficking and genetic defects in autophagy processes are among the new and most remarkable groups of neurometabolic diseases, and therefore elucidation of their pathophysiology is of great importance.

Recently, Gershlick et al. revealed compound heterozygous mutations (c.1468C>T/p.Asp745Thrfs*93 and c.2232delC/p.Arg490Cys) in the gene encoding the common GARP/EARP subunit VPS51 in a 6-year-old patient with severe global developmental delay, microcephaly, hypotonia, epilepsy, cortical visual impairment, pontocerebellar abnormalities, failure to thrive, liver dysfunction, lower extremity edema, and dysmorphic features were identified by exome sequencing. This study showed that the frameshift mutation caused by the c.2232delC mutation produces a longer but highly unstable protein, whereas the c.1468C>T mutation produces a stable protein that associates less efficiently with other GARP/EARP subunits. The levels of fully assembled GARP and EARP complexes were reduced in skin fibroblasts from the patient. The distribution of the cation-independent mannose 6 phosphate receptor was altered, and some of the patient’s fibroblasts exhibited swelling in lysosomes. With these findings, a new genetic locus for a neurodevelopmental disorder has been identified, emphasizing the critical importance of GARP/EARP function in cellular and organismal physiology [9].

Similarly, whole exome sequencing in two siblings with brain malformations consisting of cerebellar atrophy in the oldest sibling and hypoplastic corpus callosum in the younger sibling affected with delayed psychomotor development, speech deficit, severe intellectual disability, and postnatal microcephaly revealed a homozygous intragenic deletion in *VPS51* (c.1419_1421del; p. Phe474del), but no further functional studies were performed [30].

The novel homozygous missense mutation (c.1511C>T; p. Thr504Met) in the *VPS51* gene detected by exome sequencing in siblings with microcephaly, thin corpus callosum, hearing loss, developmental delay, epilepsy and swallowing disorder reported in this article supports the pathogenic effect of *VPS51* variants previously reported in the literature by Gershlick et al. and Uwineza et al. In this study, both mRNA expression and VPS51 protein expression were found to decrease as a result of examining the effect of the novel mutation detected in the *VPS51* gene at the RNA/protein level using patient fibroblast cells. VPS51 protein expressions were decreased in patients compared to the control, as previously shown in the literature [9].

Since the GARP complex contributes to pre-autophagosomal structure and the lysosome is the final stop of autophagy, we also studied key autophagy markers known as p62 (SQSTM1) and LC3B. p62 mRNA expression levels was decreased, while LC3B mRNA expression was increased in the patients. We also investigated mRNA expression level for RAB7, which mediates membrane fusion, and found that total RAB7A mRNA expression levels were not highly reduced. However, there was a halving of TBC1D15 mRNA expression level, which mediates membrane separation by hydrolyzing RAB7-GTP at the mitochondria–lysosome membrane contact [31].

In contrast to the increased levels of p62 and LC3 protein levels in the previous study in Saccharomyces cerevisiae by Perez-Victoria et al., our study showed a decrease in p62 and LC3 protein levels in patients with the *VPS51* gene defect encoding the GARP complex subunit [32]. Although the p62 mRNA expression level decreased, the LC3 mRNA expression level increased, and autophagy seemed to be increased, while the expression levels of both proteins decreased. The decrease in the total protein levels of LC3 and p62 in GARP-deficient cells may be due to the induction but not progression of the formation of autophagic structures. Proteomic analysis also showed that p62 (SQSTM1) protein expression was decreased in the patient sample compared to the control. To the best of our knowledge, this is the first study in which autophagic markers were examined in patients’ fibroblasts carrying *VPS51* gene mutation.

Furthermore, this study aimed to identify target protein(s) and associated pathways associated with vesicular traffic, which have not been previously elucidated by comparative proteome analysis.

Proteomic analysis results revealed at least a 1.5-fold difference in vesicular traffic-related proteins between patients and control samples; LRP1, TUBA4A, LMAN1, TMED2, RAB10, SEC23A, RAB1B, TUBB6, HSP90AA1, AP2A1, ACTR1A, SURF4, OPTN, TXNDC5, YWHAQ, TFRC, ARF1, RAB31, RAB18, and MYO1C were downregulated; and GOLGA5, COL1A1, DCTN3, CAPZA1, ARCN1, USO1, RAB7A, PRKAG1, SPTBN1, CAPZA2, CTTN, PICALM, SEC31A, PAFAH1B1, MCFD2, AGFG1, ARPC4, GDI1, CAPZB, PAFAH1B3, AP2S1, and ACTB were upregulated. Most of these proteins were vesicle coat, membrane, and RAB GTPase proteins. In addition, proteins detected in pathways related to mitochondrial function, such as TCA cycle, pentose phosphate pathway, fatty acid beta oxidation, and pyruvate metabolism, were found to be decreased in patients compared to control. In a study conducted in Parkinson’s patients linked to *GBA1* (β-glucocerebrosidase), a lysosomal hydrolase, it was reported that there was no significant change in RAB7 total protein levels, but TBC1D15 (RAB7-GAP) levels changed. However, further studies have shown that the RAB7-GTP/total RAB7 ratio increased. In our study, no information was obtained about TBC1D15 levels at the protein level, but RAB7 protein levels were found to increase [33]. The fact that TBC1D15 did not show a significant change in proteomics, despite the increase in Rab7 and membrane contact sites, may be related to the fact that the function of this protein is provided by activity modulation or local dynamics rather than a quantitative change. In addition, the cell may have favored alternative mechanisms to regulate the increased activities of Rab7. Further studies on the specific post-translational modifications and localization of TBC1D15 may help to better understand these results. However, in light of the present data, it appears that lysosomal and mitochondrial activities are impaired and also affect intermembrane junctions.

These changes reflect a coordinated cellular response to the *VPS51* mutation, with metabolic reprogramming in favor of survival over efficiency. The observed lipid accumulation may result from suppressed β-oxidation, while increased glycolysis and FASN activity may help to counteract energy deficits [24]. In support of this finding, changes in protein localization suggest that cellular compartments are reorganized in response to the *VPS51* mutation. Down-regulation of lysosomal, mitochondrial, and vesicle-associated proteins may indicate impaired trafficking and organelle dynamics. In contrast, up-regulation of proteins associated with the extracellular space, membrane, and Golgi apparatus may reflect compensatory mechanisms to maintain cellular homeostasis under stress conditions.

A considerable number of protein have been observed to downregulate associated with energy metabolism, cytoskeleton, stress response, and protein-folding pathways. It is hypothesized that disparities in fibroblast size and morphology may be indicative of modulated cytoskeletal organization. Fibrotic fibroblasts frequently manifest enhanced actin stress fibers and cytoskeletal remodeling in response to mechanical cues [34]. Moreover, an imbalance in the autophagy and mitophagy pathways has been identified as a contributing factor to fibrotic phenotypes. An analysis of patient-derived fibroblasts reveals the presence of mitochondrial fragmentation and functional decline, which are concomitant with morphological alterations [35].

Decreased p62 induces activation of the autophagy pathway through the Nrf2/Keap1/p62 pathway. According to the results of the proteome analysis, it is observed that the KEAP1/NRF2 pathway is downregulated in the patient. The decrease in the levels of proteins involved in this pathway and p62 protein expression suggests activation of the Nrf2/Keap1/p62 pathway [36]. Decreased antioxidant proteins (CAT, PRDX2, TXN) may indicate that cells are vulnerable to oxidative stress. Both the reduction of p62 and the reduction of proteins in the NRF2/KEAP1 pathway lead to a more severe weakening of cellular defense mechanisms and cause autophagy mechanisms to collapse. This results in susceptibility of the cell to toxic accumulation, inability to control oxidative stress, and increased mitochondrial dysfunction [37,38]. The data obtained as a result of proteomic analysis in fibroblast cells should be interpreted takinginto account the minimum sample size. However, we believe that this study, which addresses many different perspectives and is the first of its kind to study the EARP/GARP complex, is necessary and valuable for both research, clinical, and relevant patient communities.

In our study, intracellular vesicular traffic, immune system, signal transduction, L1CAM interaction, and lysosomal activity pathways were found to be affected, similar to a previous study conducted with mucolipidosis, a lysosomal storage disease [39].

**Figure 8:**
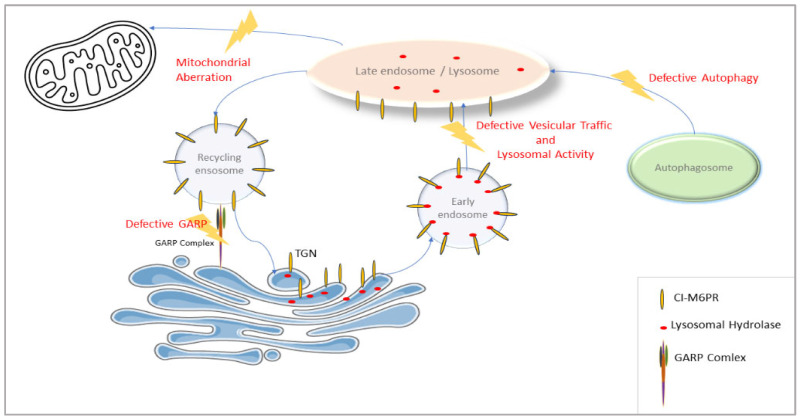
Pathological pathway in VPS51 protein defect. Impaired GARP/EARP function affects lysosomal activity and autophagy and leads to impaired vesicle-mediated transport and mitochondrial aberration.

VPS51 is involved in the regulation of a wide range of lysosomal processes, as it encodes the GARP complex subunit for the retrieval of endosomes, carrying receptors that transport lysosomal hydrolases back to trans-Golgi and the EARP complex subunit for the return of transferrin receptors from the cell membrane. The effect of reducing the activity of this gene may be more far-reaching than that seen, for example, in a specific lysosomal storage disease. Thus, the VPS51 defect leads to more far-reaching alterations in lysosomal function (supported in this study by changes in the levels of proteins in numerous lysosomal and vesicular pathways along with defective autophagy) (Figure 8).

The findings indicate that the *VPS51* mutation enhances communication between mitochondria and lysosomes, yet concomitantly leads to defects in the retromer complex and endosomal recycling mechanisms. Further functional studies are required to elucidate the mechanisms of lysosomal adaptation and mitophagy. The study of vesicular traffic and autophagy genes revealed that vesicular transmission is impaired by the *VPS51* mutation, prompting increased vesicular traffic to regulate the accumulation of proteins and reduce cell stress. Among these genes, *VPS29*, *SNX6*, and *RAB7A* have been identified as particularly significant. The VPS29 is a component of the retromer complex, which is involved in the process of retrograde transport to new organelles, especially in endosomes. RAB7A is a Rab GTPase protein that plays an important role in endosomal transport pathways and regulates vesicular transport and fusion processes to direct the retromer complex to the correct location. SNX6 (Sorting Nexin 6) is a sorting nexin protein bound to the retromer complex and is involved in cargo selection. The combined function of retromer, nexin, and RAB7A ensures the proper functioning of transduction between endosomes and lysosomes in the cell and the return of receptors to the Golgi [40,41]. Studies detailing the intracellular roles of Rab7A and VPS29 will provide a more comprehensive understanding of the mechanistic effects of the *VPS51* mutation. Although further studies are being conducted, since this is the first article in this context with membrane tethering protein, the data obtained are mostly consistent with the studies conducted in the literature with lysosomal storage and lysosomal storage-like diseases [39,42]. In addition, findings that can also be evaluated as Golgipathies were obtained (Golgi-stress response) [43].

In the context of energy metabolism, the proteins involved are of particular significance, including the enzymes that are interconnected in pathways such as the TCA cycle, glycolysis, and lipogenesis (e.g., ACLY, FASN, PGK1, IDH1, and ACO2). Further studies on these enzymes may provide valuable insights into the complex structure of disease pathophysiology. It is hypothesized that the cell may be experiencing a combined vesicular trafficking disorder, with a switch in energy production affecting pathways such as oxidative phosphorylation and glycolysis. In the event of oxidative phosphorylation being ineffective, alternative pathways, such as glycolysis and lipogenesis, may be favored.

## 4. Materials and Methods

### 4.1. Subjects

Peripheral venous blood in EDTA anticoagulant tubes were collected from each participant that clinically evaluated patients and other family members (n = 5) at Hacettepe University, Faculty of Medicine, Department of Pediatrics Metabolism. Skin biopsy samples were taken from patients with mutations in the *VPS51* gene for further functional studies. Informed consent was obtained for all of the participated individuals according to protocols approved by the Ethical Review Board Hacettepe University, (GO-15/210;GO 21/99) at Hacettepe University, Faculty of Medicine, Ankara, Turkey.

### 4.2. Genetic Investigations

#### 4.2.1. Whole Exome Sequencing

Genomic DNA was extracted from peripheral blood using a standard salting-out method [44]. Whole exome sequencing was performed for two affected patients. DNA libraries were prepared using the SureSelect Human All Exon V5 Kit according to the manufacturer’s instructions (Agilent, Santa Clara, CA, USA). Samples were sequenced on the Illumina HiSeq 4000 platform with a 150 bp paired-end strategy. Barcoded samples were sequenced in parallel, and at least 20 sequence depths per base were achieved in more than 98% of targeted regions. The quality of the reads was pre-checked with FastQC, and Trimmomatic tools was used to trim low-quality sequences and remove the adapter [45].

#### 4.2.2. Bioinformatic Analyses of Exome Sequence Data

Paired-end sequence data were aligned to the human reference genome hg19 using a Burrows–Wheeler aligner (BWA) v0.7.17. The Picard tool was used to remove PCR duplicates [46]. Variant calling was performed using GATK v3.7, and variants were functionally annotated with ANNOVAR (https://wannovar.wglab.org, accessed on 1 June 2021). The variant list obtained via data analysis was filtered according to total read depth, minor allele frequency, coding, and non-synonymous criteria. After first-line filtering, reads were checked using Integrative Genomics Viewer (IGV) [47]. Finally, prediction programs (CADD, MutationTaster, SIFT4G) were used to evaluate the damaging and pathogenicity of variants according to ACMG criteria [48].

#### 4.2.3. Mutation Analysis

Sanger sequencing of the *VPS51* gene was performed in all family members, including two patients, the parents, and one healthy sibling to confirm the variant detected by exome sequencing analyses. The PCR products were purified with the MinElute 96 UF PCR purification kit (Qiagen Inc., Valencia, CA, USA), and DNA sequencing was performed by direct sequencing of the purified PCR products using the BigDye Terminator Cycle Sequencing kit (version 3.1) and ABI 3130 automated DNA sequencer (Applied Biosystems, CA, USA). The chromatograms evaluated based on a reference sequence (GenBank: NM_013265.4) of the gene using sequencing Analysis Software v 5.2 Patch 2 (Applied Biosystems, CA, USA).

#### 4.2.4. Gibbs Free Energy Calculation and Protein Modeling of VPS51 Missense Variant

Since the VPS51 model for Homo sapiens is not available in the RCSB Protein Data Bank (https://www.rcsb.org/structure/4BX9, accessed on 1 June 2021), the protein structure for the residue of interest (Thr504) was modeled by in silico methods. The Pdb file used in the modeling was obtained using the AlphaFold prediction database (https://alphafold.ebi.ac.uk/entry/Q9UID3, accessed on 1 June 2021). We used DynaMut2 software (https://biosig.lab.uq.edu.au/dynamut2/nma, accessed on 1 June 2021), which evaluates the effects of single and also multiple missense variants on protein stability, to calculate the changes in Gibbs free energy between wild-type and altered amino acid structure [49].

#### 4.2.5. Fibroblast Cell Culture

Skin biopsy samples were taken from patients with mutations in the VPS51 gene, and fibroblast cells were grown in a culture medium. Fibroblast cells were brought to passage four and used. Proliferation of cells and passaging were performed by adding 10 mL of medium containing 90% DMEM, 10% FBS, and 1% penicillin–streptomycin to the cells seeded in T75 flasks. Cells were cultured at 37 °C, 5% CO_2_, and the medium was changed every three days to ensure complete confluency.

#### 4.2.6. RNA İsolation, cDNA Synthesis

RNA and protein isolations were first performed from fibroblast cells reaching P4 stage. Total RNA was isolated from patient and control fibroblast cells using an RNA isolation kit (Thermo Sci., PureLink RNA Mini Kit, Cat No: 12183018A, CA, USA). The density and A260/280 ratios of RNA samples obtained from each fibroblast sample using 5 × 10^6^ cells were determined using a NanoDrop ND-1000 (NanoDrop Technologies, Wilmington, NC, USA) spectrometer. All RNAs were stored at −80 °C. Total RNAs obtained from patients and control fibroblasts were used to obtain cDNA for RT-PCR experiments. cDNA synthesis was conducted on ice using a High Capacity cDNA Reverse Transcription kit (Thermo Sci., Cat No: 4368813, Applied Biosystems, Foster City, CA, USA) according to the manufacturer’s protocol. In this PCR-based method, 2 µg of RNA was prepared for each reaction and converted to cDNA. All cDNAs were stored at −20 °C until RT-PCR experiments.

#### 4.2.7. RT-PCR for mRNA Expression Studies

Maxima SYBR Green/ROX was utilized with 50 ng of cDNA for each experimental replicate. The reaction conditions were determined through a series of optimization experiments conducted for each primer. The reaction was set up in PCR microplates, with each well containing cDNA from a separate sample, and was performed with all primers at 60 °C and 48 cycles (denaturation, annealing, and extension). Two replicates were performed for each sample, and the experiment was conducted using a Stratagene MX3005P instrument (Agilent Technologies, Santa Clara, CA, USA). The results were normalized to β-actin, and Ct values were evaluated using the 2^–∆∆Ct^ method, which is a simple formula used in order to calculate the relative fold gene expression of samples.

#### 4.2.8. Protein Isolation and Western Blotting

Total protein was obtained by treating fibroblast cells grown in T75 flasks from each of the patient and healthy control samples with RIPA lysis buffer (Santa Cruz Biotechnology, Cat. No: sc-24948, Santa Cruz, CA, USA). Protein isolation was performed on ice according to the manufacturer’s protocol. The concentration of the proteins obtained was determined by a microplate reader using BSA (Bovine Serum Albumin) protein standard and Bradford method [50]. All proteins obtained for use in Western blotting and proteomics experiments were stored at −80 °C.

Proteins were isolated from primary fibroblast cultures of patients and control subjects were first loaded onto SDS–polyacrylamide gels of appropriate density (10–14%) according to their molecular weight and run in vertical gel electrophoresis. The separated proteins were transferred to membranes by semi-dry transfer (Bio-rad system). After incubation with primary antibodies and secondary antibodies of the relevant proteins (VPS51-PA558869, p62-MA527800, and LC3B-PA116930), the bands of the relevant proteins were visualized via a chemiluminescence method [51,52]. The signal was detected using a Gbox chemi XRQ imaging system (syngene). Loading controls were performed by incubating the same membranes with β-actin antibodies. The optical densities of the bands in all images were evaluated, and all bands were normalized to the optical density of β-actin. Control and experimental groups were compared for the protein marker of interest using ImageJ 1.46v software [53]. 

#### 4.2.9. Proteomics

Proteins obtained from patient fibroblast samples were detected by nano-LC-MS/MS (liquid chromatography–mass spectrometry/mass spectrometry) and fragment ion analysis method [54]. After the proteins were digested with trypsin in gel fractions and the peptides formed were cleaned by reverse phase chromatography, they were analyzed via nLC-MS/MS (Q-Exactive Orbitrap Mass Spectroscopy) Mass Spectrometry analytical system and proteins, and modified residues were reported using Mascot, Sequest, and Protein Pilot database search programs integrated into the system.

Total protein samples obtained from patient and control fibroblast cells (5 × 10^6^) were analyzed with three replicates each. Raw data were processed using MaxQuant proteomics software version 2.6.8.0 and the Andromeda plugin using the LFQ analysis method. The “Protein Group” file obtained from the raw data was matched with homo sapiens protein group data using Perseus software version 2.1.4.0. For proteins that differed between patient and control, statistically significant ones were separated using the “multiple-sample ANOVA” test. Finally, they were sorted according to which sample the difference originated from using the “Posthoc Tukey’s HSD” test. The resulting protein list was grouped by pathway and biological function using the STRING version 12.0 database.

#### 4.2.10. Live-Cell Confocal Microscopy

Patient and control fibroblasts were seeded on 96-well opaque plates at a density of 20 × 10^4^ cells per well. The plates were then incubated at 35 °C and 5% CO_2_ for 48 h, after which the cells were allowed to adhere to the plate surface and become confluent. Following this incubation period, the medium was removed, and the cells were treated with 200 µL of Mitotracker Green FM at a concentration of 100 nm in the dark. Following a 30 min incubation at 35 °C in a 5% CO_2_ atmosphere, the Mitotracker was removed, and 200 µL of Lysotracker Red DND-99 at a concentration of 200 nm was added. The plate was then incubated for a further 30 min at 35 °C in a 5% CO_2_ atmosphere. At the conclusion of the incubation period, all dyes were removed, and 200 µL of PBS was added. Confocal imaging was then performed directly in the plate in PBS containing medium. Cells were imaged with a 63x objective using a laser scanning confocal microscope Zeiss Ism980 model (Hacettepe, Ankara, Turkey)and Zeiss Zen 3.11 program.

The resulting images were analyzed with the DiAna plugin of the Fiji-ImageJ program [55]. The results obtained were statistically tested using Phyton program 3.13.1 and R program 4.4.2. In the statistical analysis, firstly, the Shapiro–Wilk test was performed in to determine whether the data showed a normal distribution. According to the distribution results, Mann–Whitney U (non-parametric) and Student’s *t*-test (parametric) were used to assess significance, and Cohen’s d test was used to determine effect size [56].

## Figures and Tables

**Table 1:** In silico predictions of possible effects of the variant detected in the *VPS51* gene and ACMG classification.

	CADD Score	SIFT4G	Mutation Taster	DynaMUT2	ACMG
VPS51; p.Thr504Met; c.1511C>T	25.6	0.01 Damaging	0.9979 Disease causing	−0.26 kcal/mol (destabilizing)	VUS

## Data Availability

The raw data supporting the conclusions of this article will be made available by the authors on request.

## Supplementary Materials

The following supporting information can be downloaded at https://www.mdpi.com/article/10.3390/ijms26125709/s1.
