# Supplementary material for: From Gene to Pathways: Understanding Novel Vps51 Variant and Its Cellular Consequences
Source: Int J Mol Sci. 2025 Jun 14;26(12):5709. doi: 10.3390/ijms26125709 (PMC12193522; doi:10.3390/ijms26125709)
Supplement: Supplementary file 1 [file ijms-26-05709-s001.zip › ijms-3675676-supplementary.pdf]

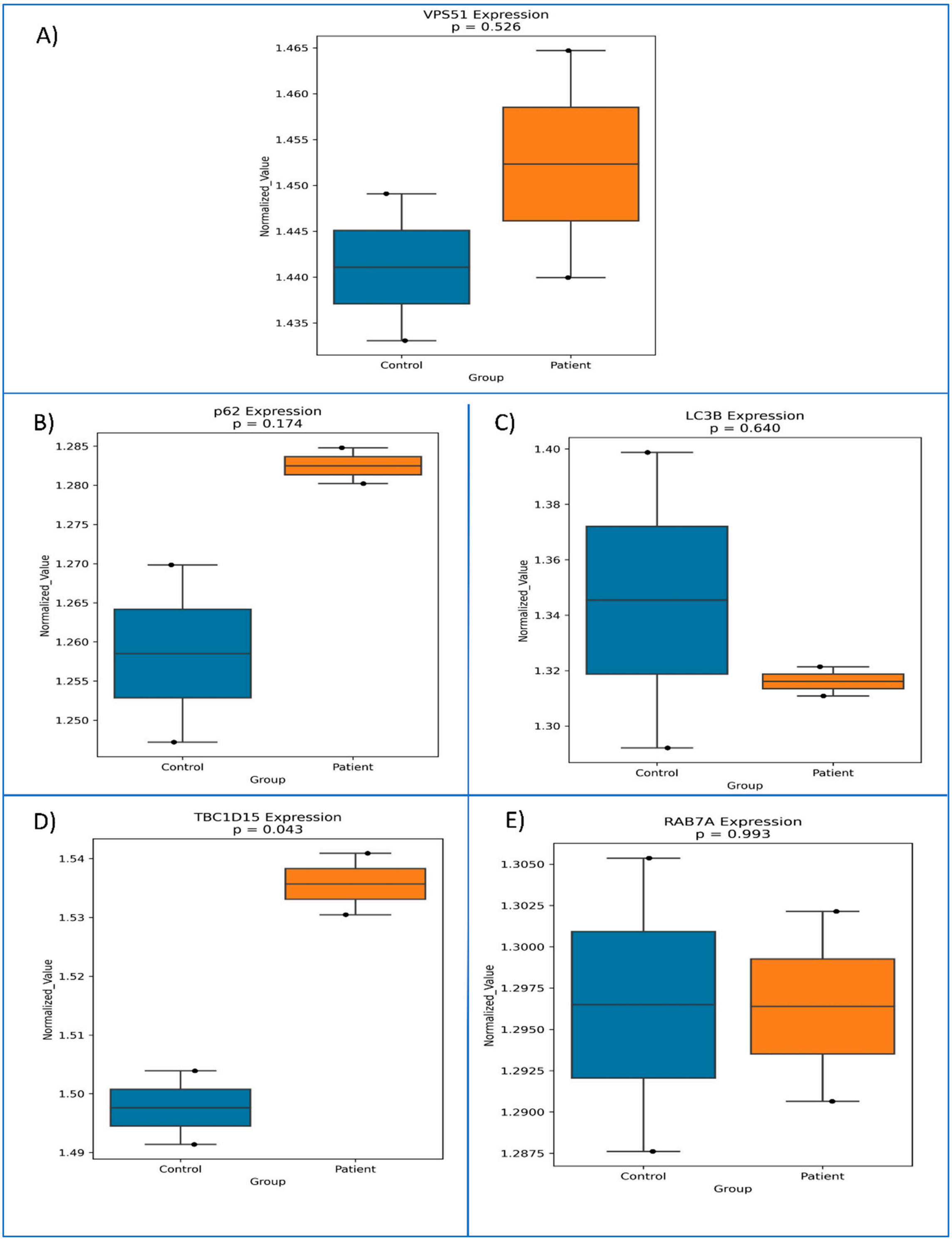

Figure S1. The following figure illustrates the boxplot of Ct values obtained from RT-PCR, which have been normalized to actin. VPS51 A), p62 B), LC3B C), TBC1D15 D), RAB7A E). The following study will examine the differences in gene expression between the patient and the control group. The dots represent individual technical replicates (n=2 per sample), and the horizontal line indicates the mean. It should be noted that no statistical analysis was performed due to the absence of independent biological replicates.

Table S1. Downregulated proteins in VPS51 deficient fibroblasts. FC: Fold change, changes are indicated by * for a protein that was detected only in the patient sample (P/C: Patient/Control, FDR<0.05).

Metabolism of proteins		Cellular responses to stress		Immune System		KEAP1-NRF2 pathway		Signal Transduction	
Gene Name	FC (P/C)	Gene Name	FC (P/C)	Gene Name	FC (P/C)	Gene Name	FC (P/C)	Gene Name	FC (P/C)
AARS1	-2,00	ACTR1A	-2,10	ACTR1A	-2,10	CSNK2A1	-2,00	AP2A1	-2,00
ACTR1A	-2,10	BAG2	-1,54	ANPEP	-2,00	DPP3	-2,03	CARM1	-2,00
ANPEP	-2,00	CARM1	-2,00	AP2A1	-2,00	G6PD	-2,00	CCT2	-2,00
ARF1*	-1,87	CAT	-2,00	ARF1*	-1,87	IDH1	-2,00	CDC42	-2,00
CCT2	-2,00	CRYAB	-2,00	CAT	-2,00	PSMA2	-2,00	COL6A3	-2,00
CCT5	-2,00	CSNK2A1	-2,00	CCT2	-2,00	PSMB2	-2,00	CSNK2A1	-2,00
CD109	-2,00	DNAJA2	-2,03	CDC42	-2,00	PSMC1	-2,00	GNB1	-2,00
CETN2	-2,00	DPP3	-2,03	CPNE1	-2,00	PSMC3	-2,00	GNB2	-2,00
CSNK2A1	-2,00	G6PD	-2,00	EEF2	-2,00	PSMD12	-2,00	HSP90AA1	-2,00
CUL4A	-2,69	HSP90AA1	-2,00	EIF2AK2	-2,00	PSMD14*	-1,68	ITGA5	-2,00
DDX17	-2,00	IDH1	-2,00	FSCN1	-1,71	PSMD6	-2,00	ITPR3	-2,00
DPP3	-2,03	MAPK3	-1,88	GBP1	-2,87	SQSTM1*	-2,14	KTN1	-2,00
EEF2	-2,00	PRDX2*	-1,95	GRN	-2,36	TXNRD1	-2,05	LAMB2	-2,03
EIF3D	-2,00	PRDX5	-2,39	GSTO1	-2,14	Apoptosis		LMAN1*	-1,79
EIF5	-1,91	PSMA2	-2,00	HSP90AA1	-2,00	Gene Name	FC (P/C)	MAPK3	-1,88
EIF5A	-2,00	PSMB2	-2,00	IDH1	-2,00	DNM1L	-2,00	MMP2	-2,04
EPRS1	-2,00	PSMC1	-2,00	IFI16	-2,00	KPNB1	-2,00	MYH10	-2,00
GBA	-2,00	PSMC3	-2,00	ITPR3	-2,00	MAPK3	-1,88	NCKAP1	-2,00
GNB1	-2,00	PSMD12	-2,00	KPNB1	-2,00	PSMA2	-2,00	OPTN	-2,00
GNB2	-2,00	PSMD14*	-1,68	LRRFIP1	-2,00	PSMB2	-2,00	PDGFRB	-2,00
KARS1	-2,00	PSMD6	-2,00	MAPK3	-1,88	PSMC1	-2,00	PELP1	-2,00
KTN1	-2,00	PTGES3	-2,00	MMP2	-2,04	PSMC3	-2,00	PHB	-2,00
LAMB2	-2,03	RPL10	-2,00	MRC2	-2,00	PSMD12	-2,00	PPP1CA	-2,00
LARS1	-2,00	RPL10A	-2,00	MTAP	-2,00	PSMD14*	-1,68	PSMA2	-2,00
LMAN1*	-1,79	RPL23A	-1,98	MYO1C	-2,00	PSMD6	-2,00	PSMB2	-2,00
MMP2	-2,04	RPL27	-2,61	NCKAP1	-2,00	UACA	-2,00	PSMC1	-2,00
NPM1	-2,00	RPS13	-2,00	PSMA2	-2,00	YWHAQ	-2,00	PSMC3	-2,00
PPA1	-2,00	RPS15A	-2,00	PSMB2	-2,00	Cell Cycle, Mitotic		PSMD12	-2,00
PSMA2	-2,00	RPS17	-2,00	PSMC1	-2,00	Gene Name	FC (P/C)	PSMD14*	-1,68
PSMB2	-2,00	RPS19	-2,00	PSMC3	-2,00	ACTR1A	-2,10	PSMD6	-2,00
PSMC1	-2,00	RPS24	-2,28	PSMD12	-2,00	CETN2	-2,00	PTBP1	-2,00
PSMC3	-2,00	RPS5	-2,00	PSMD14*	-1,68	CSNK2A1	-2,00	PTGES3	-2,00
PSMD12	-2,00	RPSA	-2,00	PSMD6	-2,00	HSP90AA1	-2,00	SHMT2	-2,00
PSMD14*	-1,68	SQSTM1*	-2,14	RAB10*	-1,74	KPNB1	-2,00	SQSTM1*	-2,14
PSMD6	-2,00	STIP1	-2,00	RAB18*	-1,50	MAPK3	-1,88	STIP1	-2,00
QARS1	-2,00	TUBA4A	-2,00	RAB31	-2,68	OPTN	-2,00	TFRC	-2,00

Metabolism of proteins		Cellular responses to stress		Immune System		Cell Cycle, Mitotic		Signal Transduction	
Gene Name	FC (P/C)	Gene Name	FC (P/C)	Gene Name	FC (P/C)	Gene Name	FC (P/C)	Gene Name	FC (P/C)
RAB10*	-1,74	TUBB6	-2,00	SEC23A	-2,00	PSMA2	-2,00	TMED2*	-2,03
RAB18*	-1,50	TXN	-2,00	SQSTM1*	-2,14	PSMB2	-2,00	TUBA4A	-2,00
RAB1B	-2,21	TXNRD1	-2,05	SRP14	-1,79	PSMC1	-2,00	TUBB6	-2,00
RAB31	-2,68	XPO1	-2,00	SURF4	-1,95	PSMC3	-2,00	UACA	-2,00
RCN1	-2,30	Vesicle-mediated transport		TRIM25	-2,25	PSMD12	-2,00	XPO1	-2,00
RPL10	-2,00	Gene Name	FC (P/C)	TUBA4A	-2,00	PSMD14*	-1,68	YWHAQ	-2,00
RPL10A	-2,00	LMAN1*	-1,79	TUBB6	-2,00	PSMD6	-2,00	Metabolism of lipids	
RPL23A	-1,98	LRP1	-2,00	TXN	-2,00	RAB1B	-2,21	Gene Name	FC (P/C)
RPL27	-2,61	MYO1C	-2,00	TXNDC1	-2,05	TUBA4A	-2,00	ACADM	-2,00
RPS13	-2,00	OPTN	-2,00	XRCC6	-2,00	TUBB6	-2,00	ARF1*	-1,87
RPS15A	-2,00	RAB10*	-1,74	Translation		XPO1	-2,00	CARM1	-2,00
RPS17	-2,00	RAB18*	-1,50	Gene Name	FC (P/C)	Mitochondrial protein import		CPNE1	-2,00
RPS19	-2,00	RAB1B	-2,21	AARS1	-2,00	Gene Name	FC (P/C)	CSNK2A1	-2,00
RPS24	-2,28	RAB31	-2,68	EEF2	-2,00	ACO2	-2,00	DECR1	-2,00
RPS5	-2,00	SEC23A	-2,00	EIF3D	-2,00	CS*	-1,57	ECHS1	-2,00
RPSA	-2,00	SURF4	-1,95	EIF5	-1,91	PITRM1	-2,00	FDPS	-2,00
SEC23A	-2,00	TFRC	-2,00	EPRS1	-2,00	SLC25A13	-2,00	GBA	-2,00
SQSTM1*	-2,14	TMED2*	-2,03	KARS1	-2,00	Citric acid cycle (TCA cycle)		GPD2	-2,00
SRP14	-1,79	TUBA4A	-2,00	LARS1	-2,00	Gene Name	FC (P/C)	HSD17B4*	-1,84
SRP68	-2,00	TUBB6	-2,00	PPA1	-2,00	ACO2	-2,00	KPNB1	-2,00
SUMF2	-2,23	TXNDC1	-2,05	QARS1	-2,00	CS*	-1,57	PPP1CA	-2,00
TBCB	-2,00	YWHAQ	-2,00	RPL10	-2,00	FH	-1,81	PTGES3	-2,00
THY1	-2,00	ACTR1A	-2,10	RPL10A	-2,00	Mitochondrial fatty acid beta-oxidation		SEC23A	-2,00
TMED2*	-2,03	AP2A1	-2,00	RPL23A	-1,98	Gene Name	FC (P/C)	SUMF2	-2,23
TRIM25	-2,25	ARF1*	-1,87	RPL27	-2,61	ACADM	-2,00	TXNRD1	-2,05
TRMT112	-2,00	HSP90AA1	-2,00	RPS13	-2,00	DECR1	-2,00		
TUBA4A	-2,00	L1CAM interactions		RPS15A	-2,00	Autophagy			
TUBB6	-2,00	Gene Name	FC (P/C)	RPS17	-2,00	Gene Name	FC (P/C)		
TXN	-2,00	AP2A1	-2,00	RPS19	-2,00	ATG3	-2,00		
UGGT1	-2,00	CSNK2A1	-2,00	RPS24	-2,28	CSNK2A1	-2,00		
		ITGA5	-2,00	RPS5	-2,00	HSP90AA1	-2,00		
		MAPK3	-1,88	RPSA	-2,00	SQSTM1*	-2,14		
		TUBA4A	-2,00	SRP14	-1,79	TUBA4A	-2,00		
		TUBB6	-2,00	SRP68	-2,00	TUBB6	-2,00		
				TRMT112	-2,00				

Table S2. Upregulated proteins in VPS51 deficient fibroblasts. FC: Fold change, changes are indicated by * for a protein that was detected only in the patient sample (P/C: Patient/Control, FDR<0.05).

Metabolism of proteins		Signal Transduction		Immune System		Vesicle mediated transport		Cellular responses to stress	
Gene Name	FC (P/C)	Gene Name	FC (P/C)	Gene Name	FC (P/C)	Gene Name	FC (P/C)	Gene Name	FC (P/C)
ACTB	1,97	ACTB	1,97	ACLY	2,00	ACTB	1,97	ATOX1	2,48
ARCN1	2,39	AP2S1	2,04	ACTB	1,97	AGFG1*	1,61	CAMK2D	2
CAPZA1	2,00	ARPC4*	1,70	ALDOA	1,82	AP2S1	2,04	CAPZA1	2
CAPZA2	2,00	ATP2A2	2,00	ANXA2	2,08	ARCN1	2,02	CAPZA2	2
CAPZB*	1,61	CAMK2D	2,00	AP2S1	2,04	ARPC4*	1,70	CAPZB*	1,61
CCT4	2,00	CAPZB*	1,61	ARPC4*	1,70	CAPZA1	2,00	DCTN3	2
DCTN3	2,00	CAVIN1	2,00	ATOX1	2,48	CAPZA2	2,00	HSBP1	2
DDB1	2,00	COL1A1	2,09	CAMK2D	2,00	CAPZB*	1,61	P4HB	2
EEF1B2	2,00	COL5A2	2,00	CAPZA1	2,00	COL1A1	2,09	PDIA5	2,69
EEF1D	2,08	COL6A2	2,00	CAPZA2	2,00	CTTN	2,00	PRDX6	2,00
GNG12	2,00	CTTN	2,00	CAPZB*	1,61	GDI1	2,00	PSMA5	2,61
IARS2	2,20	EMD	1,71	CD63	2,00	GOLGA5	2,00	PSMD10*	1,56
KIF13A	2,00	FASN	2,00	COL1A1	2,00	MCFD2	2,00	PSMD7	2,11
LMCD1	2,00	FKBP1A	1,70	DCTN3	2,00	PAFAH1B1	2,36	RPL18	2,59
MCFD2	2,00	FLNA	2,00	FKBP1A	1,70	PAFAH1B3	2,76	RPL21	2,26
MFGE8	2,00	GNG12	2,00	FLNA	2,00	PICALM	2,00	RPS21	2,00
P4HB	2,00	HNRNPM*	1,81	FLNB	2,00	PRKAG1	2,00	SEC31A*	1,99
PARK7	1,88	HSPE1	2,00	ICAM1	2,00	RAB7A	1,55	Extracellular matrix organization	
PSMA5	2,61	LETM1	2,48	KRT1	2,00	SEC31A*	1,99	Gene Name	FC (P/C)
PSMD10*	1,56	MOSPD2	2,00	LTA4H	2,06	SPTBN1	2,00	COL12A1	2,00
PSMD7	2,11	P4HB	2,00	MOSPD2	2,00	USO1	2,26	COL1A1	2,09
RAB7A	1,55	PAFAH1B1	2,36	P4HB	2,00	Translation		COL5A2	2,00
RPL18	2,59	PICALM	2,00	PRDX4	1,89	Gene Name	FC (P/C)	COL6A2	2,00
RPL21	2,26	PRKAG1	2,00	PRDX6	2,00	EEF1B2	2,00	EMILIN1	2,00
RPN1	2,00	PSAP	2,00	PSAP	2,00	EEF1D	2,08	FBLN1	2,20
RPS21	2,00	PSMA5	2,61	PSMA5	2,61	IARS2	2,2	ICAM1	2,00
RUVBL1	2,13	PSMD10*	1,56	PSMD10*	1,56	RPL18	2,59	P3H1	2,00
SEC31A*	1,99	PSMD7	2,11	RAB7A	1,55	RPL21	2,26	P4HB	2,00
SPTBN1	2,00	RAB7A	1,55	SEC31A*	1,99	RPN1	2,00	TNC	2,00
ST3GAL2	2,00	RACK1	2,40	STOM	2,00	RPS21	2,00		
STT3A	1,76	RUVBL1	2,13	PSMD7	2,11	TUFM	2,00		
TGFBI	1,81	SPTBN1	2,00	PTPN23	2,33				
TNC	2,00	STOM	2,00						
TUFM	2,00	VPS29	2,00						
USO1	2,26								
VDAC2*	1,76								

Table S3. Molecular function of downregulated proteins of patient.

term description	observed gene count	matching proteins
Protein binding	107	GRN,SNRPD3,EIF5,CSNK2A1,MMP2,DECR1,NAMPT,TRIM23,EIF2AK2,CAT,LRP1,TUBA4A,LMAN1,SHFL,UGGT1,PSMC1,PDGFRB,MAPK3,CNRIP1,RAB10,PRDX5,CCT5,ATG3,THY1,CD109,KPNB1,ITGA5,MAP1B,NPM1,CCT2,PELP1,HINT1,GNB2,LAMB2,QARS1,EEF2,SRP68,HNRNPD,DNAJA2,GBA,CPNE1,NCL,TRIM25,KARS1,CARM1,PPP1CA,SHMT2,NEXN,HSP90AA1,EIF5A,NME1,SND1,STIP1,AP2A1,H2AC11,XRCC6,MYH10,FH,EPRS1,IFI16,HTRA1,CETN2,GBP1,BAG2,ACADM,SERBP1,TXN,CUL4A,GNB1,OPTN,ASPH,IPO7,YWHAQ,FSCN1,SQSTM1,LRRFIP1,TFRC,INF2,G6PD,ANXA4,KTN1,XPO1,RPL23A,RPSA,IDH1,SEPTIN9,SLC25A13,UBE2L3,MPST,HSD17B4,RPS24,SSBP1,CRYAB,TXNRD1,TRMT112,ARF1,PHB2,DNM1L,RPS19,PHB,SH3GLB1,PSMC3,PTGES3,MYO1C,CDC42,SUMF2,SNX6
Catalytic activity	95	PLOD1,ACO2,CSNK2A1,APMAP,MMP2,DECR1,NAMPT,PLOD3,TRIM23,EIF2AK2,SPR,CAT,UGGT1,PSMC1,AARS1,PDGFRB,MAPK3,RAB10,PRDX5,CCT5,ATG3,CCT2,ANPEP,PRDX2,PPIC,HINT1,GNB2,QARS1,EEF2,GPD2,RAB1B,GBA,POGLUT3,FKBP10,CPNE1,TRIM25,KARS1,CARM1,PPP1CA,SHMT2,HSP90AA1,NME1,CS,SND1,FDPS,ATP5F1C,XRCC6,OGA,FH,EPRS1,ECHS1,HTRA1,GSTO1,GBP1,ACADM,PPA1,TXN,GNB1,ASPH,TXNDC5,PITRM1,AK3,GART,SQSTM1,G6PD,LARS1,DDX17,HSDL2,P4HA2,SMS,PSMD14,SCRN1,IDH1,SEPTIN9,UBE2L3,PAPSS2,MPST,HSD17B4,TXNRD1,TRMT112,ARF1,DPP3,NCEH1,DNM1L,SQOR,RAB31,CAPNS1,RAB18,EPHX1,PSMC3,PTGES3,PHGDH,MTAP,HK1,CDC42
Ion binding	82	RCN1,PLOD1,ACO2,EIF5,CSNK2A1,MMP2,DECR1,PLOD3,TRIM23,EIF2AK2,CAT,LRP1,TUBA4A,LMAN1,PSMC1,AARS1,PDGFRB,MAPK3,RAB10,CCT5,KPNB1,ITGA5,CCT2,ANPEP,SEC23A,QARS1,EEF2,GPD2,RAB1B,DNAJA2,FKBP10,CPNE1,TUBB6,TRIM25,KARS1,FHL5,PPP1CA,SHMT2,HSP90AA1,NME1,FDPS,XRCC6,MYH10,EPRS1,ACTR1A,CETN2,GBP1,ACADM,PPA1,ITPR3,OPTN,ASPH,PITRM1,AK3,GART,SQSTM1,ANXA4,LARS1,DDX17,P4HA2,PSMD14,DYSF,IDH1,SEPTIN9,SLC25A13,UBE2L3,PAPSS2,CRYAB,TXNRD1,ARF1,DPP3,NCEH1,DNM1L,SQOR,RAB31,CAPNS1,RAB18,PSMC3,HK1,MYO1C,CDC42,SUMF2
Hydrolase activity	42	APMAP,MMP2,TRIM23,CAT,PSMC1,AARS1,RAB10,CCT5,CCT2,ANPEP,HINT1,GNB2,EEF2,RAB1B,GBA,CPNE1,PPP1CA,HSP90AA1,NME1,SND1,XRCC6,OGA,HTRA1,GBP1,PPA1,GNB1,PITRM1,LARS1,DDX17,PSMD14,SCRN1,SEPTIN9,ARF1,DPP3,NCEH1,DNM1L,RAB31,CAPNS1,RAB18,EPHX1,PSMC3,CDC42
Enzyme binding	39	SNRPD3,CAT,TUBA4A,PDGFRB,MAPK3,ATG3,THY1,KPNB1,NPM1,CCT2,PELP1,HINT1,GNB2,QARS1,EEF2,HNRNPD,NCL,PPP1CA,HSP90AA1,AP2A1,H2AC11,EPRS1,GBP1,BAG2,CUL4A,GNB1,OPTN,IPO7,SQSTM1,TFRC,INF2,XPO1,UBE2L3,DNM1L,RPS19,PHB,PTGES3,MYO1C,CDC42
Cell adhesion molecule binding	19	EIF5,RAB10,THY1,ITGA5,LAMB2,EEF2,TRIM25,PPP1CA,NEXN,SND1,SERBP1,FSCN1,LRRFIP1,KTN1,RPL23A,RPSA,IDH1,SEPTIN9,SH3GLB1
GTPase activity	13	TRIM23,RAB10,GNB2,EEF2,RAB1B,GBP1,GNB1,SEPTIN9,ARF1,DNM1L,RAB31,RAB18,CDC42
Unfolded protein binding	9	LMAN1,UGGT1,CCT5,NPM1,CCT2,DNAJA2,HSP90AA1,CRYAB,PTGES3
Ligase activity	8	AARS1,QARS1,TRIM25,KARS1,ATP5F1C,EPRS1,GART,LARS1
Antioxidant activity	6	CAT,PRDX5,PRDX2,GSTO1,TXN,TXNRD1
NADP binding	5	DECR1,SPR,CAT,G6PD,IDH1

Table S4. Molecular function of upregulated proteins of patient.

term description	observed gene count	matching proteins
Protein binding	84	HSPB6,GOLGA5,PSMD10,PSMD7,COL1A1,HSPE1,PHPT1,KRT1,KIF13A,IPO5,CCDC6,CAPZA1,CAPG,ICAM1,USO1,RAB7A,TNC,PTPN23,MFGE8,CDH13,SETD7,STOM,ETHE1,ADH5,ARFIP1,DDB1,FASN,CSPG4,RUVBL1,CD276,PRKAG1,MYO1D,HNRNPM,FLNC,P4HB,FBLN1,PARK7,PRDX6,RPS21,ANXA2,SPTBN1,CAVIN1,TPM1,CALD1,CAPZA2,CSRP1,CACYBP,EMD,FLNA,GNG12,COL5A2,CTTN,ACOT7,AHNAK,PRDX4,EMILIN1,TAGLN,PICALM,ST3GAL2,CCT4,PSAP,SEC31A,PAFAH1B1,FKBP1A,ARPC4,CHCHD3,HSBP1,MYO1A,GDI1,CAPZB,EEF1D,SERPINE2,TGFBI,NUDT5,FLNB,CAMK2D,RACK1,WDR1,ATOX1,ATP2A2,PAFAH1B3,AP2S1,ACTB,ALDOA
Cell adhesion molecule binding	27	CAPZA1,CAPG,ICAM1,USO1,MFGE8,CDH13,FASN,RUVBL1,P4HB,FBLN1,PARK7,PRDX6,ANXA2,SPTBN1,CALD1,EMD,FLNA,CTTN,AHNAK,EMILIN1,PICALM,CAPZB,EEF1D,TGFBI,FLNB,RACK1,ALDOA
Cytoskeletal protein binding	27	KIF13A,CAPZA1,CAPG,MYO1D,FLNC,P4HB,ANXA2,SPTBN1,TPM1,CALD1,CAPZA2,CSRP1,CACYBP,EMD,FLNA,CTTN,TAGLN,PICALM,PAFAH1B1,ARPC4,MYO1A,CAPZB,FLNB,CAMK2D,WDR1,ACTB,ALDOA
